# USP22 maintains gastric cancer stem cell stemness and promotes gastric cancer progression by stabilizing BMI1 protein

**DOI:** 10.18632/oncotarget.16445

**Published:** 2017-03-22

**Authors:** Yue Ma, Hua-Lin Fu, Zhen Wang, Hai Huang, Jian Ni, Jie Song, Ying Xia, Wei-Lin Jin, Da-Xiang Cui

**Affiliations:** ^1^ Institute of Nano Biomedicine and Engineering, Shanghai Engineering Center for Intelligent Diagnosis and Treatment Instrument, Department of Instrument Science and Engineering, Key Laboratory for Thin Film and Microfabrication Technology of Ministry of Education, School of Electronic Information and Electronic Engineering, Shanghai Jiao Tong University, Shanghai 200240, China; ^2^ School of Biomedical Engineering, Shanghai Jiao Tong University, Shanghai 200240, China; ^3^ National Center for Translational Medicine, Collaborative Innovational Center for System Biology, Shanghai Jiao Tong University, Shanghai 200240, China; ^4^ Center for Mitochondrial Biology and Medicine, The Key Laboratory of Biomedical Information Engineering of Ministry of Education, School of Life Science and Technology and Frontier Institute of Science and Technology, Xi’an Jiao Tong University, Xi’an 710049, China; ^5^ Department of Clinical Biochemistry, School of Clinical Laboratory Science, Guizhou Medical University, Guiyang, Guizhou 550005, China

**Keywords:** USP22, BMI1, gastric cancer stem cell, gastric cancer, deubiquitinase

## Abstract

Increased ubiquitin-specific protease 22 (USP22) has been associated with poor prognosis in several cancers including gastric cancer. However, the role of USP22 in gastric tumorigenesis is still unclear. Gastric cancer stem cells have been identified and shown to correlate with gastric cancer initiation and metastasis. In this study, we found that silencing of USP22 inhibited proliferation of gastric cancer cells and suppressed the cancer stem cell spheroid formation in serum-free culture. Furthermore, cancer stem cell markers, such as CD133, SOX2, OCT4 and NANOG were down-regulated. Additionally, knockdown of USP22 inhibited gastric cancer xenografts growth. Our analysis of TCGA database indicated that BMI1 overexpression may predict gastric cancer patient survival, and TAT-BMI1 proteins reversed the USP22 knockdown-mediated decreased in cancer stem cell properties, and elevated the expression of stemness-associated genes. Furthermore, we found that overexpression of USP22 stabilized the BMI1 protein in gastric cancer cells. Taken together, our study demonstrates that USP22 is indispensable for gastric cancer stem cell self-renewal through stabilization of BMI1. These results may provide novel approaches to the theranostics of gastric cancer in the near future.

## INTRODUCTION

Gastric cancer (GC) is a common malignancy worldwide with high death mortality and low cure rates. Despite advances in surgical treatment and chemotherapy for GC, the prognosis for advanced GC is still very poor [1, 2]. Therefore, elucidation of the mechanisms underlying GC and development of new treatment strategies are urgently needed.

Cancer stem cells (CSCs) are a subpopulation of cells capable of self-renewal and unlimited replication to initiate tumors and have been well-characterized in multiple malignancies [3, 4]. CSC theory proposes that CSCs are the major cause of tumor recurrence due to their resistance to traditional radiotherapy and chemotherapy [5]. Researchers have identified stem cell markers for various cancers. Gastric CSCs were detected, isolated and shown to express increased levels of CD44, CD133, OCT4, SOX2, GLI1, p-AKT and p-ERK [6–8]. However, the knowledge of the generation and regulation of gastric CSCs is still unclear, and elucidation of the mechanisms underlying gastric CSC induction is vital for GC diagnosis and treatment.

Eleven Polycomb/stem cells genes, including *USP22* were identified as death-from-cancer signatures from transgenic mouse models and cancer patients and could predict poor therapeutic outcome in multiple cancers [9, 10]. The eleven gene signatures were *GBX2*, *KI67*, *CCNB1*, *BUB1*, *KNTC2*, *USP22*, *HCFC1*, *RNF2*, *ANK3*, *FGFR2* and *CES1*, which are involved in the BMI1 pathway [9]. The conserved BMI1-driven Polycomb signature regulates stemness in both tissue stem cells and CSCs [11]. USP22 is a member of the largest subfamily of deubiquitinases (DUBs) family named ubiquitin-specific proteases (USPs), which can reverse the process of ubiquitination of H2A and H2B, and some non-histone proteins [12, 13]. Meanwhile USP22 is also a conserved component of the human SAGA (Spt-Ada-Gcn5-acetyltransferase) transcriptional complex and potential CSC marker. It regulates gene transcription for cell-cycle progression [14–16] and has been reported as a potential oncogene in many cancers, including lung, prostate, cervical and hepatocellular cancers [17–19]. USP22 expression is correlated with cancer progression and tumor invasion, and had synergistic effects with C-myc in GC tissues. Meanwhile, USP22 and BMI1 co-activation may be associated with GC progression and poor prognosis [20]. Little is known about the pathogenic mechanisms of USP22 involved in GC tumorigenesis.

In this study, we analysed eleven death-from-cancer signatures in survival of cancer patients and demonstrated that USP22 plays vital roles in gastric CSC self-renewal and GC progression by stabilizing BMI1 and regulating the expression of stemness genes such as CD133, SOX2, OCT4 and NANOG.

## RESULTS

### USP22 silencing inhibits GC cells proliferation

**Figure 1:**
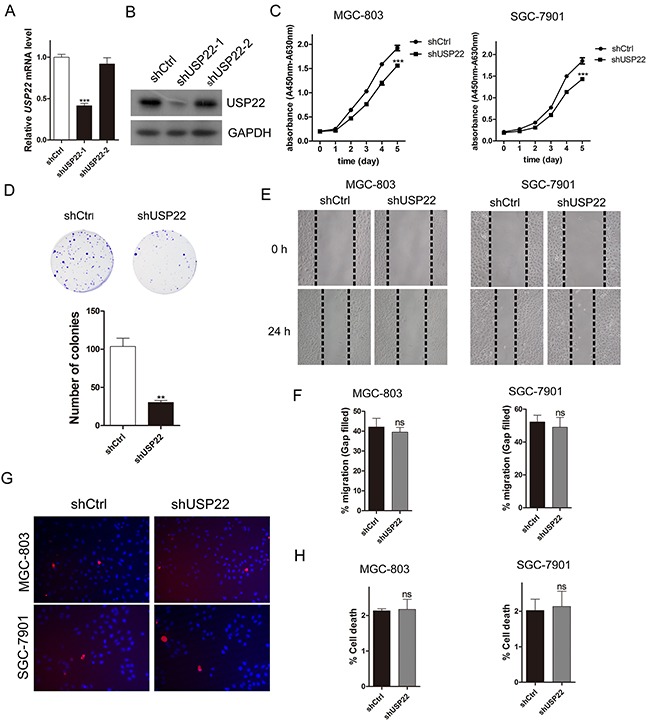
Knockdown of USP22 inhibits GC cell proliferation MGC-803 cells were infected with lentiviral vectors expressing shCtrl (Control) or shUSP22-1# and 2# (shRNAs for USP22 knockdown). **(A)** RT-qPCR validation of USP22 knockdown efficiency. **(B)** Western blot analysis of USP22 expression. GAPDH was used as a loading control. **(C)** Cell proliferation was measured using wst-1 assays. **(D)** Effect of USP22 depletion on cell colony formation. After 10 d of infection, the cells were fixed and stained with crystal violet solution. The histogram shows the colony number. **(E)** MGC-803 and SGC-7901 cells were infected with shCtrl or shUSP22 lentiviral vector. After 72 h of infection, the scratch wound-healing assay was performed to study the effect of USP22 on cell migration. The results are shown in **(F)**. **(G)** PI/Hoechst staining was conducted to evaluate the effect of USP22 on cell death. **(H)** The histograms representing the cell death percentage in **(G)**. The data were from three independent experiments. Statistical comparisons between groups were conducted by unpaired Student's t-test. Bar graph shows the mean± SEM. Statistical significance: **P<0.01, ***P<0.001, compared with control. *P*<0.05 was considered to be significant.

To investigate the role of USP22 in GC progression, we designed two shRNAs sequences (shUSP22-1, shUSP22-2). The two kinds of USP22 KD shRNAs and the control shRNA were cloned into lentiviral vector. The knockdown efficiency was confirmed in GC MGC-803 cells by both RT-qPCR and Western blot analyses, and shUSP22-1 was shown to be more effective (Figure 1A–1B).

We performed cell proliferation assays of MGC-803 and SGC-7901 cells using wst-1 reagent (Figure 1C). The results showed that cell proliferation was significantly inhibited in USP22-silenced cells. Meanwhile, the colony formation assays of MGC-803 cells also revealed that USP22 knockdown suppressed the proliferative ability of GC cells (Figure 1D).

Wound-healing assays were carried out in MGC-803 cells and SGC-7901 cells to verify the effect of USP22 depletion on cell migration. There was no difference in migration distance between the control and the knockdown (KD) group, suggesting that USP22 does not affect cell migration (Figure 1E–1F).

We next explored whether USP22 depletion affects GC cell apoptosis. PI/Hoechst staining exhibited little difference between the control and USP22 KD group both in MGC-803 and SGC-7901 cells (Figure 1G–1H). Taken together, these results demonstrate that knockdown USP22 predominantly affects cell proliferation instead of cell migration and apoptosis in the GC cell lines MGC-803 and SGC-7901.

### USP22 is dispensable for gastric CSC formation and stemness maintenance

**Figure 2:**
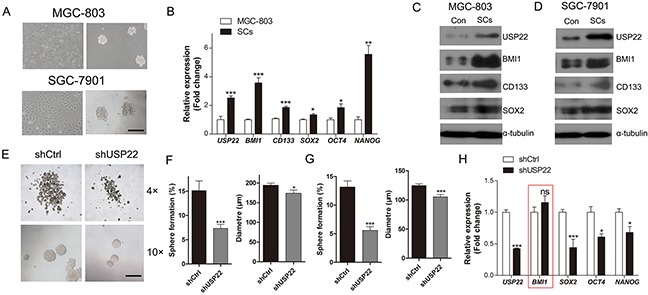
Inhibitory effect of USP22-silencing on gastric CSC formation **(A)** Cultured gastric CSCs derived from GC cell lines MGC-803 and SGC-7901 cells in serum-free culture. Scale bar=100 μm. **(B)** RT-qPCR analysis of the gastric CSC markers in MGC-803 cells and the MGC-803 derived stem cells (SCs). **(C-D)** Western blot analysis of the expression of gastric CSC markers in SCs and control. α-tubulin was chosen as endogenous control. **(E)** The effect of USP22 depletion on gastric CSC formation in MGC-803 and SGC-7901 cells in serum-free culture. **(F)** Histograms show the stem cell spheroid formation and the sizes of the spheres. **(G)** The stem cell spheroids in **(E) (F)** were passaged 2 times, and the percentage of spheroid formation and the sizes of the spheres were calculated. **(H)** RT-qPCR analysis of the expression of gastric CSC markers in control (shCtrl) and USP22 knockdown (shUSP22) cells. Data are presented as mean±SEM. Statistical comparisons between groups were conducted by unpaired Student's t-test. Statistical significance: *P<0.05, **P<0.01, ***P<0.001, compared with the control. *P*<0.05 was considered to be significant.

Because USP22 was identified as a CSC marker and regulates the progression and prognosis of multiple cancers [10, 14], and CSCs are responsible for cancer initiation and metastasis, we explored the effect of USP22 on GC stem-like cell (SC) formation and GC progression. We isolated the spheroid cells from the GC cell lines MGC-803 cells and SGC-7901 cells in serum-free culture; these cells have stem cell-like properties, suggesting that the spheres are composed of CSCs or SCs as previously reported [21] (Figure 2A). We demonstrated that the isolated CSCs were enriched in stem cell markers, such as USP22, BMI1, CD133 and SOX2, expressed high levels of KI67, and had the capacity for self-renewal (Supplementary Figure 1). Meanwhile the RT-qPCR results showed increased mRNA expression of *USP22*, *BMI1*, *CD133*, *SOX2*, *OCT4* and *NANOG* in SCs to that of serum-cultured MGC-803 cells (Figure 2B). Additionally, the protein levels of USP22, BMI1, CD133 and SOX2 were higher in SCs than those in serum-cultured MGC-803 cells and SGC-7901 cells (Figure 2C–2D).

We next studied the effect of USP22 on gastric stem cell-like properties. We investigated the effects of USP22 silencing on stem cell-like properties using serum-free cultured CSCs. The percentage of sphere formation and sphere size were calculated at DIV 7 (Figure 2E). Notably, USP22-depleted cells showed a substantial decrease in stem cell sphere-forming ability (Figure 2F). Similar results were obtained when the sphere cells were passaged 2 times, indicating that knockdown of USP22 suppressed the self-renewal of CSCs (Figure 2G). These findings revealed that knockdown of USP22 inhibited CSC formation and stemness maintenance. The RT-qPCR results showed that USP22 silencing decreased the mRNA levels of stem cell markers, such as *SOX2*, *OCT4* and *NANOG*, but surprisingly, the expression of *BMI1* was not changed (Figure 2H). These data indicated that knockdown of USP22 suppresses the stem cell-like properties of GC cells.

### Knockdown of USP22 suppresses GC xenografts growth

**Figure 3:**
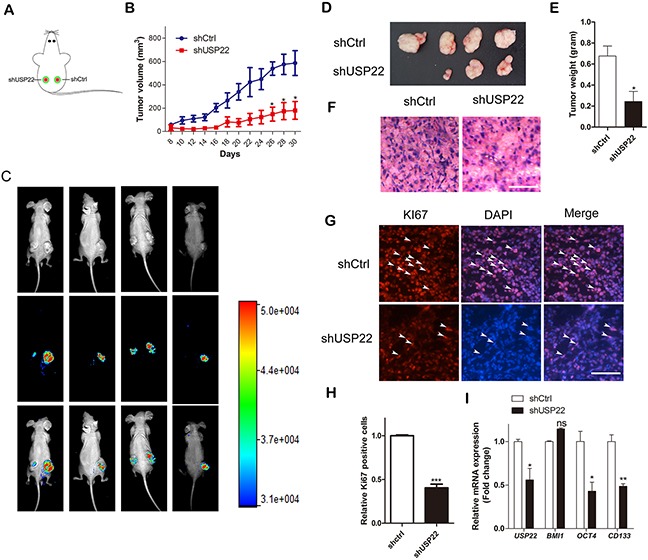
USP22 silencing suppresses tumor growth in GC xenografts *in vivo* **(A)** Male 4-week-old BALB/c mice were subcutaneously inoculated into two hind flanks with stably expressed GFP-tagged shCtrl or shUSP22 cells. **(B)** Tumor volumes were calculated after 8 d every other day by measuring the length and width of tumor until 30 d and plotted. **(C)** Tumor growth progression was monitored by *in vivo* imaging of the xenografts at 30 d after inoculation. **(D)** Representative photos of tumors 30 d after subcutaneous xenografting (n=4). Xenografts were weighed as shown in **(E)**. **(F)** H&E staining of the frozen sections of xenografts. Scale bar=100 μm. **(G)** Immunostaining of the frozen sections with KI67 antibody. Arrowheads indicate the KI67 positive cells. Scale bar=100 μm. **(H)** The relative KI67-positive cells were calculated, and statistical results are shown in the histogram. **(I)** RT-qPCR was performed to detect the mRNA expression of *USP22*, *BMI1* and gastric CSC markers. Data are presented as mean± SEM. Statistical comparisons between groups were conducted by unpaired Student's t-test. Statistical significance: *P<0.05, **P<0.01, ***P<0.001, compared with the control. *P*<0.05 was considered to be significant.

To assess the effect of USP22 on gastric tumorigenesis and cancer progression, we subcutaneously inoculated stable USP22-silenced USP22 MGC-803 cells (shUSP22 with GFP tag) and negative control (shCtrl with GFP tag) cells (5×10^6^) into the flanks of BALB/c mice respectively (one flank for shCtrl cells and the other for shUSP22 cells). Then, tumor growth was examined by measuring the tumor sizes every other day (Figure 3A–3B). The volumes of the tumors derived from USP22-depleted cells were smaller than than those from the shCtrl cells, especially from 26 d to 30 d. The tumors derived from USP22-silencing cells exhibited lower fluorescence intensity compared with that of the controls (Figure 3C). The tumor-bearing mice were sacrificed at 30 d, and the tumors formed from USP22-depleted cells weighed less than that of the controls (Figure 3D–3E). Hematoxylin and eosin (H&E) staining showed that the cancer cells in the control group grew well, whereas the USP22 knockdown group had large patches of necrosis in the xenografts (Figure 3F). The frequency of KI67-positive nuclear staining was substantially decreased in tumor tissues from USP22-silenced cells compared to those of the controls (30% versus 100%, respectively) (Figure 3G–3H). Down-regulated USP22 was observed in tumor tissues derived from USP22-depleted cells, with lower mRNA expression of *CD133* and *OCT4* compared to that of the tumor tissue from control cells (Figure 3I). However, the *BMI1* mRNA was not changed, which was consistent with Figure 2H. These data suggested that USP22 silencing has an inhibitory effect on gastric tumor growth and regulates stemness-associated gene expression.

### BMI1 overexpression predicts poor survival for GC patients

**Figure 4:**
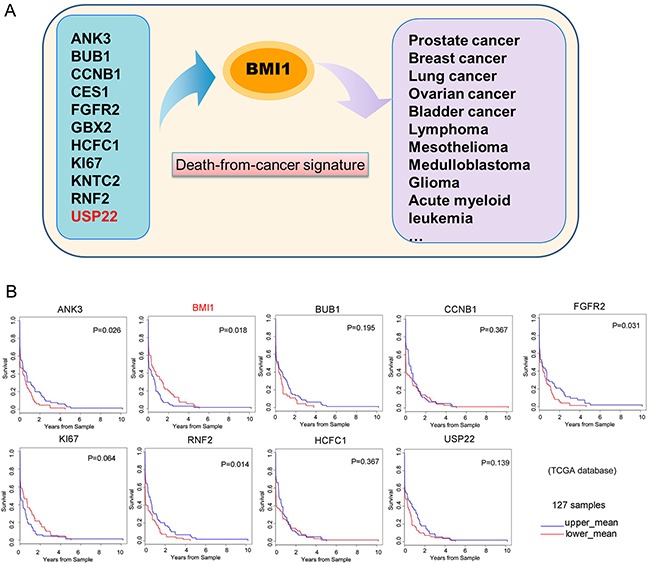
GC patient survival plots of ‘death-from-cancer’ genes **(A)** A schematic representing the prediction model for multiple cancers from death-from-cancer signatures. **(B)** GC patient survival plots of death-from-cancer genes were analysed in 127 samples from TCGA database. The GC patients were divided into two groups according to gene expression. Blue curves indicate the upper mean group, and red curves indicate the lower mean group.

Eleven genes were identified as death-from-cancer signatures predicting poor survival of cancer patients in multiple malignancies including USP22 [10] (Figure 4A). To investigate the signatures in GC, we analysed these genes in TCGA GC database. As shown in Figure 4B, elevated (upper mean group) levels of *BMI1* were associated with poor survival. These findings are consistent with previous studies showing that BMI1 (a known stem cell marker) may be a potent target for treatment of GC [6, 22]. Surprisingly, USP22, which has been reported to be elevated in multiple cancers including GC, and is prognostic for disease progression, showed no significant difference in mRNA levels in our analyses.

### BMI1 abrogates the inhibitory effect of USP22 knockdown on CSC formation

**Figure 5:**
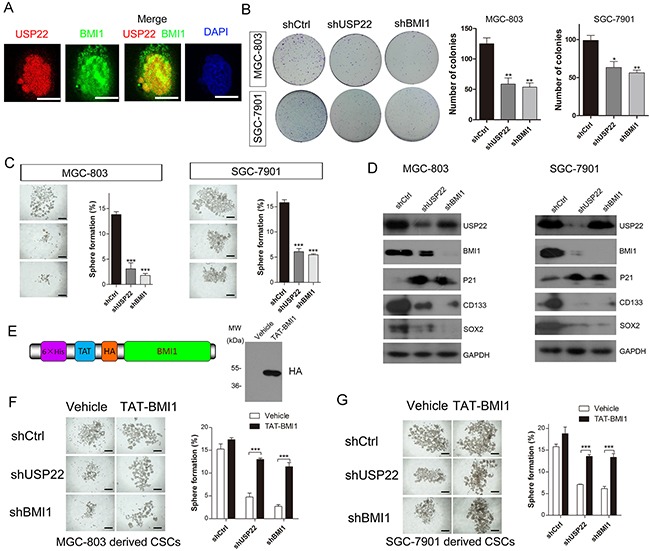
USP22 silencing inhibits gastric CS formation by regulating BMI1 protein levels **(A)** Immunofluorescence staining to analyse the co-localization of USP22 and BMI1 in MGC-803 cells. Scale bar=10 μm. **(B)** Effect of USP22 and BMI1 silencing on colony formation in MGC-803 cells and SGC-7901 cells. Colonies were calculated and statistical results are shown in histograms. **(C)** Effect of knockdown of USP22 or BMI1 on GC stem cell spheroids formation using MGC-803 and SGC-7901 cells in serum-free culture. Histograms show the percentage of spheres. **(D)** Western blot was performed to analyse the effect of USP22 and BMI1 depletion on proliferation-related and gastric SC markers using respective antibodies. GAPDH was chosen as a loading control. **(E)** Schematic of purified TAT-BMI1 protein containing a His tag, a TAT transduction domain and a HA tag (left). Validation of the transportation of BMI1 into MGC-803 cells (right). Vehicle (PBS) or TAT-BMI1 (0.05 μM) was added to cultured MGC-803 **(F)** and SGC-7901 **(G)** cells stably expressing the shCtrl, shUSP22 or shBMI1 *in vitro* sphere-forming assays. The number of gastric CSC spheres was counted and plotted relative to initial cell number (10^3^ cells) per well in a low-attachment 24-well plate. Data are presented as mean± SEM. Statistical comparisons between groups were conducted by unpaired Student's t-test. Statistical significance: *P<0.05, **P<0.01, ***P<0.001, compared with control. *P*<0.05 was considered to be significant.

Previous studies have shown that the eleven gene signatures involved in the BMI1 pathway regulate stemness in both tissue stem cells and CSCs [9, 11, 23, 24]. In colorectal carcinoma, USP22 promoted cell proliferation by activating BMI1-mediated INK4a/ARF pathway and Akt pathway [25]. In GC, co-expression of USP22 and BMI1 was prognostic for gastric cancer progression and treatment failure [20]. Immunofluorescence staining results indicated that USP22 and BMI1 are both distributed in the nucleus and partially co-localized in MGC-803 cells (Figure 5A). Meanwhile, BMI1 was also a CSC marker. We found that both USP22 and BMI1 silencing inhibited colony growth and CSCs formation of MGC-803 and SGC-7901 cells (Figure 5B–5C). We next examined the possible molecular mechanism underlying CSC formation. In MGC-803 cells and SGC-7901 cells, knockdown of USP22 and BMI1 both increased P21 expression and reduced the expression of CSC stemness genes of CD133 and SOX2. Additionally, USP22 silencing led to down-regulated BMI1 (Figure 5D).

To investigate the role of BMI1 in gastric CSC formation, we used a TAT-mediated protein transduction technology in the stem cell sphere formation experiments [26]. The TAT protein transduction peptide could carry the recombinant proteins into cells via electrical interactions with the cell membrane. We previously demonstrated the effectiveness of this system and it is a promising approach for protein transportation [27]. In our study, BMI1 was fused with the TAT protein transduction domain, yielding TAT-BMI1 (Figure 5E left panel). TAT-BMI1 transportation was validated by Western blot analysis (Figure 5E right panel). The MGC-803 cells and SGC-7901 cells were infected with shCtrl, shUSP22 and shBMI1 lentiviral vectors and formed CSCs in serum-free medium. We found that both USP22 and BMI1 silencing impaired sphere formation both in MGC-803 and SG7-7901-derived CSCs. The inhibitory effect of USP22 or BMI1 silencing on CSC sphere formation was substantially abrogated when cells were treated with 0.05 μM TAT-BMI1 every day (Figure 5F–5G). These results indicated that TAT-BMI1 protein could significantly rescue the impaired spheres formation ability induced by USP22 and BMI1 knockdown, which shows that USP22 regulates gastric CSCs through BMI1.

### USP22 stabilizes BMI1 protein

Above, we demonstrated that USP22 silencing predominantly decreases the BMI1 protein levels rather than mRNA expression (Figure 2H, Figure 5D), and further alters GC cell proliferation, gastric CSC formation and maintenance of stem cell stemness, indicating post-transcriptional regulation of BMI1. Based on these data, we concluded that USP22 regulates BMI1 turnover.

**Figure 6:**
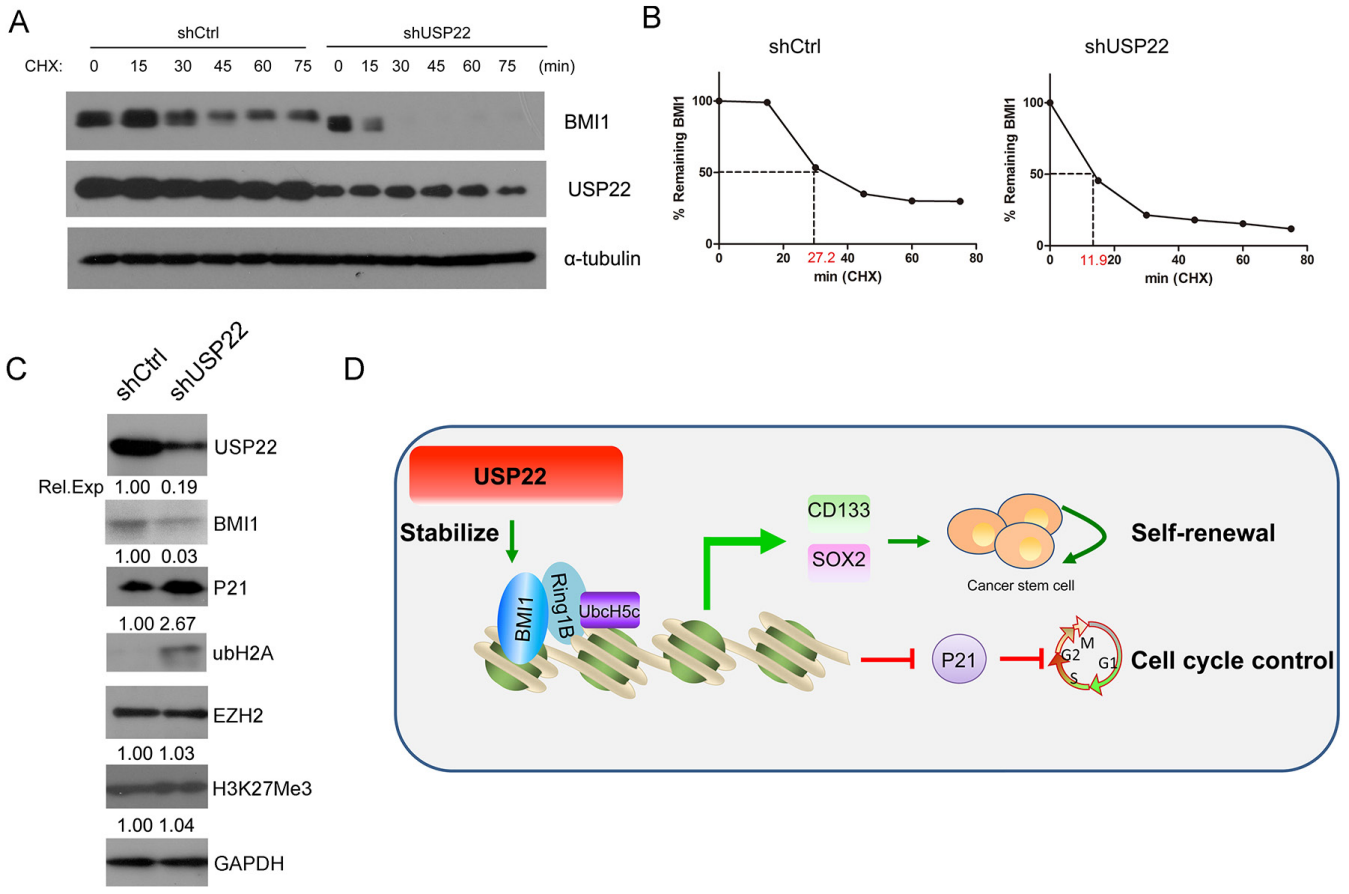
USP22 is associated with BMI1 protein stability **(A)** Stable USP22-silenced (shUSP22) or control (shCtrl) MGC-803 cells were treated with CHX (20 μM) for 0, 15, 30, 45, 60 and 75 min, and endogenous BMI1 proteins were examined by Western blot. GAPDH was used as an endogenous control. **(B)** The densitometry curves of BMI1 normalized to GAPDH were plotted against the indicated time points to determine its half-lives according to **(A)**. **(C)** Western blot was conducted to verify the effect of knockdown of USP22 on PcG protein EZH2 level and H3K27me3. GAPDH was used as a loading control. **(D)** A proposed working model of the function of USP22 in regulating gastric tumorigenesis.

A previous study had revealed that the BMI1 undergoes proteasome-mediated degradation [28]. To explore the mechanism of posttranslational regulation of BMI1, we examined the half-lives of BMI1 in lentivirus-mediated USP22-silenced and control MGC-803 cells by inhibiting de novo protein synthesis using cycloheximide (CHX) treatment for different time periods (0-75 min). Western blot analysis was performed to examine BMI1 protein levels at each time point and protein levels were quantified by densitometry (Figure 6A–6B). The results showed that the half-life of BMI1 was significantly shortened in the USP22-silenced cells compared to that of the control (27.2 min to 11 min). These data suggested that USP22 stabilizes BMI1 protein. We found USP22 silencing led to decreased expression of BMI1, as well as decreased P21 levels. Meanwhile, EZH2, another Polycomb-group (PcG) protein associated with histone modification of H3K27me3 (Figure 6C), showed no change in abundance, consistent with a study reporting that USP22 silencing did not change EZH2 in hepatocellular carcinoma [29]. Our results also confirmed that USP22 silencing caused increased ubH2A expression (Figure 6C), in accordance with a previous report demonstrating that ubH2A was a substrate of USP22 [14].

We found that USP22 silencing could stabilize BMI1 protein and BMI1 plays vital roles in self-renewal of neural stem cells, hematopoietic stem cells, mammary stem cells, and CSCs [30–32]. Based on these results, we developed a working model showing that USP22 could stabilize BMI1 and further regulate CSC origin and self-renewal (Figure 6D).

### USP22 and BMI1 overexpression is correlated with in clinicopathological characteristics and poor prognosis of GC

**Figure 7:**
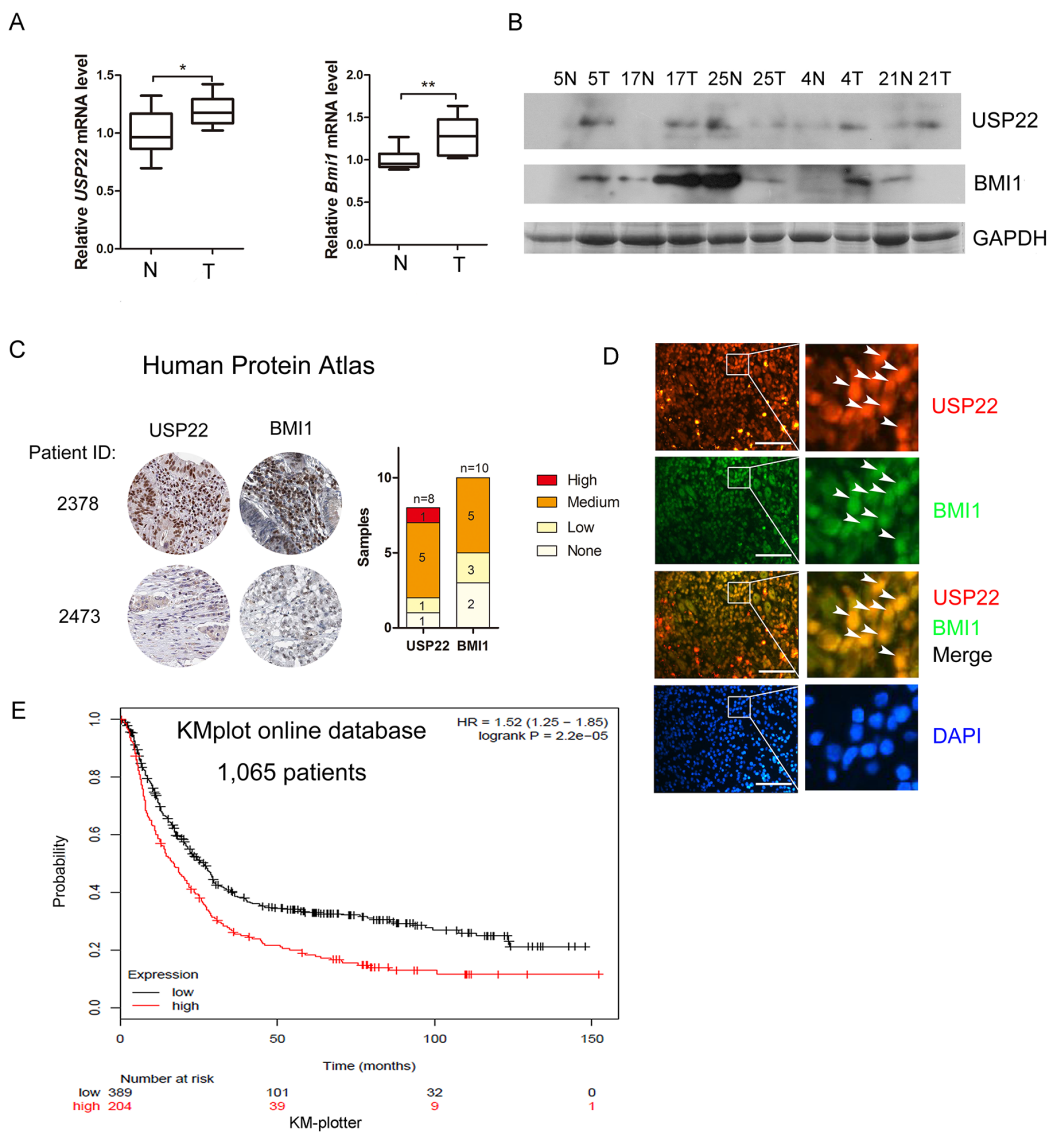
Overexpression of USP22 correlates with BMI1 in GC tissue samples and is association with poor prognosis **(A)** RT-qPCR analysis of mRNA expression of *USP22* and *BMI1* in 10 pairs of GC (T) and adjacent non-cancerous tissues (T). **(B)** Western blot to detect USP22 and BMI1 expression in GC clinical specimens. GAPDH was used as a loading control. **(C)** Representative immunohistochemistry analysis from the human protein atlas (HPA) database. USP22 and BMI1 levels were plotted according to TNM staging. **(D)** Immunofluorescence staining of GC tissue sections to analyse the expression and co-localization of USP22 and BMI1 in clinical specimens. Arrowheads indicate the USP22 positive/BMI1 positive cells. Scale bar=50 μm. **(E)** Kaplan-Meier survival plots shows USP22 and overall survival rate of 1,065 patients from the GC database (www.Kmplot.com).

We compared USP22/BMI1 expression in clinical GC (T) and normal stomach (N) tissues. As shown in Figure 7A, mRNA levels of USP22 and BMI1 were much higher in GC tissues than those in normal stomach tissues. Overexpression of USP22 was correlated with BMI1 in 3 out of 5 pairs of specimens (Figure 7B).

We also compared USP22 and BMI1 expression in specimens from the Human Protein Atlas (HPA) program databases and found a positive correlation between USP22 and BMI1 in GC. USP22 and BMI1 were positively associated with TNM staging (Figure 7C). Immunofluorescence staining of gastric cancer tissues also showed strong nuclear co-localization of USP22 and BMI1 (Figure 7D). Kaplan-Meier survival plots show that higher expression of USP22 leads to a lower overall survival rate of patients compared to those with low USP22 levels [33] (Figure 7E). Thus, overexpression of USP22 is positively associated withBMI1 in clinicopathology and correlates with poor prognosis of GC.

## DISCUSSION

A comprehensive understanding of the molecular mechanisms involved in GC initiation and progression is essential to optimize current strategies and identify new molecular-targeted treatments. Increasing evidence has shown that the USP family members are differentially expressed or elevated in tumors, indicating they are potential therapeutic targets in cancer treatment [34–38]. Although USP22 was reported as a “death-from-cancer” signature and was aberrantly increased in many cancers, the molecular mechanisms underlying the elevated expression of USP22 during GC progression and poor prognosis are still elusive [39–41]. Here, we found that USP22 is highly overexpressed in GC and positively associated with BMI1. USP22 silencing resulted in a significant reduction in the proliferation of MGC-803 and SGC-7901 cells. Meanwhile, the protein levels of BMI1 were down-regulated following USP22 knockdown. Additionally, we demonstrated that knockdown of USP22 suppresses gastric CSC formation and hinders CSC stemness. Our findings indicate for the first time that USP22 plays critical roles in maintaining gastric CSC self-renewal and GC progression through stabilization of BMI1.

The SAGA complex is composed of multiple functional modules possessing both HAT (GCN5) and deubiquitination (USP22) activities in transcription [42]. Involvement of GCN5, USP22 and other submodules of SAGA have been linked with various cancers. GCN5 was shown to be required for SAGA to activate c-Myc target genes and promotes non-small cell lung cancer, glioma and hepatocellular carcinoma progression [43–46]. USP22 was also identified as an oncogene and associated with many cancers, such as colorectal cancer, breast cancer, oral squamous cell carcinoma, anaplastic thyroid carcinoma, glioma and GC [25, 41, 47–49].

A previous study showed that USP22 is required for cell cycle progression, and USP22 deletion suppresses proliferation of multiple cancer cells [16, 50–51]. USP22 regulates P21 expression by affecting FBP1 occupy at *CDKN1A*(*P21)* gene loci [12]. We found that USP22 depletion inhibited the proliferation of the human GC cell lines MGC-803 and SGC-7901 cells and increased expression of P21, indicating cell cycle arrest [52]. However, we did not observe significant changes in cell migration and apoptosis between USP22-silenced cells and controls *in vitro*.

An increasing body of evidence supports the existence of CSCs, which possess characteristics associated with stem cells that have the ability to initiate tumor growth and sustain tumor self-renewal [53–57]. Previous studies have demonstrated that CSCs are related to drug resistance. Due to limitations in traditional cancer treatments, a new strategy to develop anti-cancer drug targeting CSCs, preventing tumor recurrence, received much attention [58]. Gastric CSCs were identified using a method known as spheroid colony formation [6, 59]. The cultured CSCs exhibited increased expression of CSC markers, such as SOX2, OCT4, NANOG and CD133, consistent with the results of immunostaining of the CSC spheres (Supplementary Figure 1). As USP22 was identified as a putative CSC marker, we found the USP22 was overexpressed in gastric CSCs. Furthermore, our results showed that USP22 depletion significantly inhibits CSCs formation.

BMI1 is a core component of the Polycomb group (PcG) proteins of epigenetic enzymes, regulating stem cell self-renewal and lineage development [60–62]. BMI1 is overexpressed in many cancers, including breast cancer, prostate cancer and GC [10, 22, 63]. The *BMI1* oncogene-driven pathway was demonstrated to be a key regulatory mechanism affecting stemness in both normal and CSCs [10, 23]. A recent study highlighted the importance of BMI1 in maintaining gastric CSCs properties [6, 22]. Our results showed that USP22 silencing significantly down-regulated BMI1 protein expression and further affected gastric CSC self-renewal. Overexpression of USP22 and BMI1 was previously associated with GC progression and therapy failure through clinical specimen analysis, and our study is consistent with these results. Here, we demonstrated that USP22 is indispensable for stabilizing BMI1 protein in the gastric CSC formation. As a member of DUBs, USP22 could stabilize multiple proteins, such as SIRT1, TRF1 and AR through deubiquitination [13, 64, 65]. Whether USP22 could stabilize BMI1 through deubiquitination requires further exploration.

In this study, we demonstrated that USP22-mediated protein stabilization of BMI1 promotes gastric CSC stemness maintenance and GC progression, thereby providing a rationale for USP22 targeting as a potential therapeutic approach against GC.

## MATERIALS AND METHODS

### Compliance with ethical standards

All protocols were in accordance with the ethical standards of the responsible committee on human experimentation (institutional and national) and with the Helsinki Declaration of 1975, as revised in 2000 (5). Informed consent was obtained from all patients prior to inclusion in the study.

All institutional and national guidelines for the care and use of laboratory animals were followed.

### Human GC xenograft experiments

Four- to six-week old male BALB/c nude mice were used for the xenograft experiments. Cancer cells were trypsinized, harvested in PBS and counted, and a total volume of 0.1 mL PBS (2×10^6^ cells) was injected subcutaneously into the flanks.

### Cell culture and GC SC culture

The human GC cell line MGC-803 cells was obtained from the Cell Resource Center, Shanghai Institute of Biochemistry and Cell Biology at the Chinese Academy of Sciences. SGC-7901 cell lines were generously gifted from Dr. Wei (First Affiliated Hospital of Nanjing Medical University). The authenticity of these cell lines was tested by short tandem repeat profiling. The cells were cultured in Dulbecco's modified eagle's medium (DMEM, Corning) supplemented with 10% fetal bovine serum (Gibco) at 37°C in a humidified 5% CO_2_ atmosphere.

The gastric CSCs were cultured as described in our previous report [21]. The CSCs were isolated from MGC-803 or SGC-7901 cells in serum free medium containing Neurobasal (Gibco), 20 μL/mL B27 supplement (Life Technologies), and 20 ng/mL EGF (Sigma). The cells formed sphere-like cell aggregates in less than 7 days.

### Cell proliferation assays

The proliferation of cells was detected using wst-1 reagent (Roche) according to the manufacturer's instructions. Briefly, cells were seeded at a density of 1×10^3^ cells/well in a 96-well plate and cultured for 0, 24, 48, 72, 96 and 120 h. Then, at the different time points, 10 μL wst-1 reagent was added to each well and incubated for 1 h. The absorbance was measured at 450 nm and 630 nm using a standard microplate reader (Scientific MultiskanMK3, Thermo Scientific, USA).

### Cell death assay

The cell death rate was evaluated by PI/Hoechst staining. Cultured cells were incubated in medium containing 5 μg/mL PI and 5μg/mL Hoechst at 37°C for 5 min. The cell death rate was calculated by determining PI(+)/Hoechst(+).

### Wound-healing assays

Wound-healing assays were conducted as previously described [21]. MGC-803 cells and SGC-7901 cells were seeded in 6-well plates and cultured to 80-90% confluence. After serum starvation for 12 h, a wound was then created by scraping the cell monolayer with a 200μL pipette tip. The cultures were washed with PBS to remove the floating cells. Then the cells were cultured in serum-free medium. Cell migration into the wound was observed at the indicated times (0 h, 24 h) in marked microscopic fields and mages were captured with a Nikon DS-5M Camera System. The data obtained were presented as a migration percentage by measuring the distances between wound edges with ImageJ software.

### Clonogenic assays

Briefly, cells were harvested, counted and plated at 1,000 cells per well in triplicate in a 6-well plate. Cells were cultured for 7-10 days or until viable colonies reached >100 cells. Colonies were stained with crystal violet (0.4% crystal violet, 20% ethanol) and counted.

### Quantitative real-time RT-PCR

Total RNA was extracted using TRIzol (Invitrogen) following the manufacturer's protocol. cDNAs were generated from 1 μg of total RNA using reverse transcriptase with random hexamer primers (Promega). Quantitative real-time RT-PCR was performed using specific primers (Supplementary Table 1) in a 20 μL reaction volume containing 10 μL 2×SYBY Green Mix (GeneCopeia) on an iQ5 system (Bio-Rad). The △Ct value from each sample was calculated by normalizing with HPRT or β-actin from triplicate experiments.

### Western blot analysis

Extracts of cells or tissues were prepared. The protein concentration was determined by BCA assay kit (Thermo Scientific). Equal amounts of proteins (30 μg) were subjected to SDS-PAGE and transferred to polyvinylidene fluoride membranes (Millipore). The membranes were treated with 1% blocking solution in TBS for 1 h, and immunoblots were probed with the indicated antibodies: USP22 (Santa Cruz), BMI1 (Abcam), CD133 (Abcam), SOX2 (Abcam), P21 (Proteintech), ubH2A (CST), EZH2 (CST), H3K27Me3 (CST), tubulin (Santa Cruz), HA (Abmart) GAPDH (Santa Cruz), and actin (Santa Cruz) at 4°C overnight. Then the membranes were washed and incubated with HRP-labelled secondary antibodies (1:5,000; Santa Cruz). The fluorescence signals were detected by a BM Chemiluminescence Western Blotting kit (Roche). Densitometry quantification was calculated and analysed using ImageJ software.

### Immunofluorescence staining and H&E staining for tissue sections

The xenografts were fixed with 4% paraformal-dehyde and dehydrated through 2 changes of 10% and 20% sucrose solutions. After the samples were embedded with opti-mum cutting temperature compound, they were cryosectioned into 8-μm slices. For tissue slice immunofluorescent staining, slides were boiled in 10 mM sodium citrate buffer, pH 6.0, and maintained at a sub-boiling temperature for 10 min. Then, they were cooled on a bench top for 30 min. The sections were washed in 1×TBST for 5 min and blocked with buffer containing 15% donkey serum for 1 h at room temperature. The blocking solution was removed and the samples were incubated at 4°C in diluted primary antibody solution overnight. After they were rinsed with PBS 3 times, the sections were incubated with PBS-diluted Alexa Fluor-labelled secondary antibodies (Molecular Probes 1:800) for 2 h at room temperature. The sections were washed three times with PBS and mounted with glycerine/PBS containing 0.1 mg/mL DAPI for nuclei staining, and the slides were covered.

For H&E staining, the sections were stained with hematoxylin for 2 min and then rinse with running tap water for 3 min. The sections were dipped into acidic alcohol for 50 s and then rinsed with running tap water for 3 min. After bluning in saturated lithium carbonate solution for 30 s followed by rinsing with running tap water, the slides were counterstained in eosin solution for 1 min, and rinsed with running tap water. Then the sections were placed in 70%, 80%, 95% and 100% ethanol in sequence for 1 min each. They were cleared in 2 washes of xylene for 10 min. The slides were mounted with neutral balsam and covered with coverslips.

### Statistical analysis

Data are expressed as mean ± SEM. Statistical comparisons between groups were conducted by unpaired Student's t-test. * indicates *p*<0.05; ** indicates *p*<0.01; and *** indicates *p*<0.001. *p*< 0.05 was considered to be statistically significant.

## SUPPLEMENTARY MATERIALS FIGURES AND TABLES



## Abbreviations

CHX: cycloheximide
CSC: cancer stem cells
DUB: deubiquitylase
GC: gastric cancer
PcG: Polycomb-group
SAGA: Spt-Ada-Gcn5-acetyltransferase
USP22: ubiquitin-specific protease 22.
