# Supplementary material for: USP22 maintains gastric cancer stem cell stemness and promotes gastric cancer progression by stabilizing BMI1 protein
Source: Oncotarget. 2017 Mar 22;8(20):33329–42. doi: 10.18632/oncotarget.16445 (PMC5464871; doi:10.18632/oncotarget.16445)
Supplement: Supplementary file 1 [file oncotarget-08-33329-s001.pdf]

USP22 maintains gastric cancer stem cell stemness and promotes gastric cancer progression by stabilizing BMI1 protein

SUPPLEMENTARY MATERIALS

SUPPLEMENTARY FIGURE AND TABLES

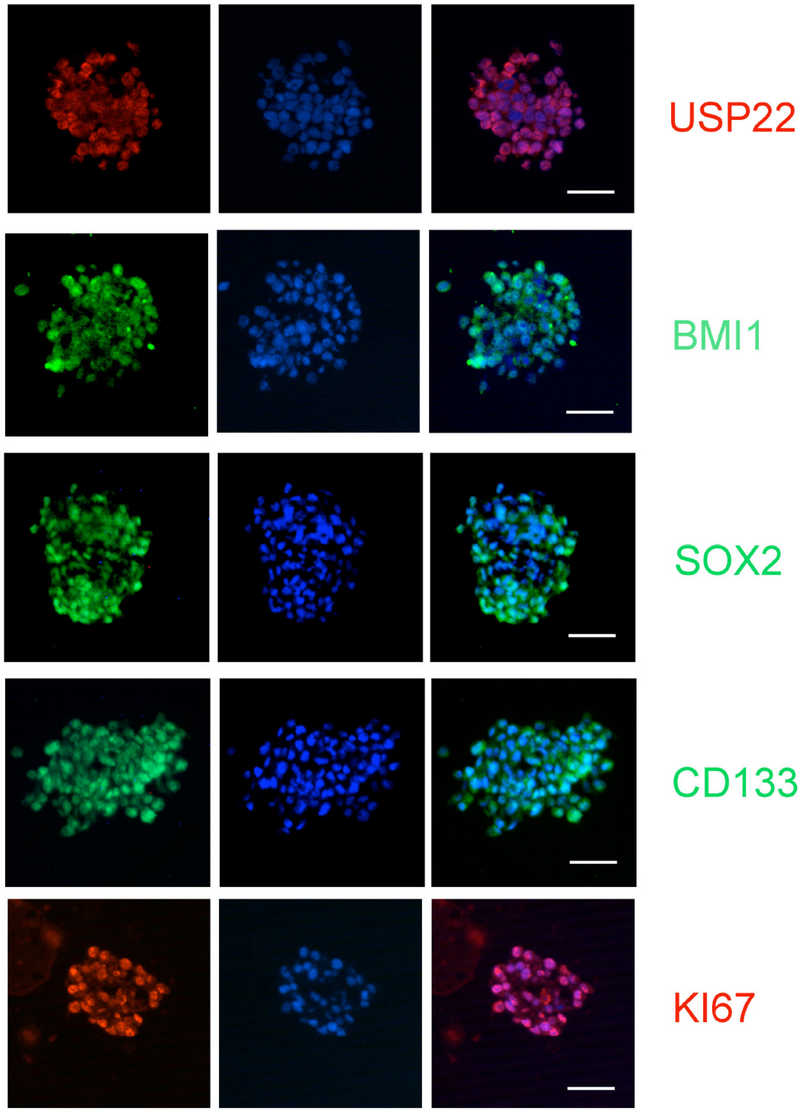

Supplementary Figure 1: Immunostaining of gastric CSCs. Immunofluorescence staining of cry-sections of gastric CSCs with indicated antibodies. Scale bar=100μm.

Supplementary Table 1: The sequences of qRT-PCR primers

Primer	Sequence (5' to 3')
BMI1 (Forward)	GGAGGAGGTGAATGATAAAAGAT
BMI1 (Reverse)	AGGTTCCTCCTCATACATGACA
USP22 (Forward)	CTCCTGTCTGGTCTGTGAGATG
USP22 (Reverse)	CAGCAACTTATACGGGATGTGA
CD133 (Forward)	ACCAGGTAAGAACCCGGATCAA
CD133 (Reverse)	CAAGAATTCCGCCTCCTAGCACT
SOX2 (Forward)	GCCCCCAGCAGACTTCACAT
SOX2 (Reverse)	AGGGGCAGTGTGCCGTTAAT
OCT4 (Forward)	GTGTTCAGCCAAAAGACCATCT
OCT4 (Reverse)	GGCCTGCATGAGGGTTTCT
HPRT1 (Forward)	CCTGGCGTCGTGATTAGTGAT
HPRT1 (Reverse)	AGACGTTCAGTCCTGTCCATAA
actin (Forward)	TCCCTGGAGAAGAGCTACG
actin (Reverse)	GTAGTTTCGTGGATGCCACA

Supplementary Table 2: The Sequences of double-strand shRNA oligonucleotides

Name	Sequence (5' to 3')
USP22-1 shRNA (sense)	GATCCGCTGTTTCACAAAGAAGCATATTC AAGAGATATGCTTCTTTGTGAAACAGCTTTTTTG
USP22-1 shRNA (antisense)	AATTCAAAAAAGCTGTTTCACAAAGAAG CATATCTCTTGAATATGCTTCTTTGTGAAACAGCG
USP22-1 shRNA (sense)	GATCCGAGCTACCAGGAGTCCACAAATT CAAGAGATTTGTGGACTCCTGGTAGCTCTTTTTTG
USP22-2 shRNA (antisense)	AATTCAAAAAAGAGCTACCAGGAGTCC ACAAATCTCTTGAATTTGTGGACTCCTGGTAGCTCG
BMI1-1 shRNA (sense)	GATCCGCCAGACCACTACTGAATATAATTC AAGAGATTATATTCAGTAGTGGTCTGGCTTTTTTG
BMI1-1 shRNA (antisense)	AATTCAAAAAAGCCAGACCACTACTGAA TATAATCTCTTGAATTATATTCAGTAGTGGTCTGGCG
BMI1-2 shRNA (sense)	GATCCGCCTAATACTTTCCAGATTGATTTC AAGAGAATCAATCTGGAAAGTATTAGGCTTTTTTG
BMI1-2 shRNA (antisense)	AATTCAAAAAAGCCTAATACTTTCCAGA TTGATTCTCTTGAAATCAATCTGGAAAGTATTAGGCG
